# Review of the Impact Pathways of Biofortified Foods and Food Products

**DOI:** 10.3390/nu14061200

**Published:** 2022-03-12

**Authors:** Samantha L. Huey, Jesse T. Krisher, Arini Bhargava, Valerie M. Friesen, Elsa M. Konieczynski, Mduduzi N. N. Mbuya, Neel H. Mehta, Eva Monterrosa, Annette M. Nyangaresi, Saurabh Mehta

**Affiliations:** 1 Division of Nutritional Sciences, Cornell University, Ithaca, NY 14853, USA; slh277@cornell.edu (S.L.H.); jtk93@cornell.edu (J.T.K.); arinibhargava.22@gmail.com (A.B.); emk285@cornell.edu (E.M.K.); nhm43@cornell.edu (N.H.M.); 2 Program in International Nutrition, Cornell University, Ithaca, NY 14853, USA; 3 Global Alliance for Improved Nutrition, 1202 Geneva, Switzerland; vfriesen@gainhealth.org (V.M.F.); emonterrosa@gainhealth.org (E.M.); 4 Global Alliance for Improved Nutrition, Washington, DC 20036, USA; mmbuya@gainhealth.org; 5 Global Alliance for Improved Nutrition, Nairobi 00800, Kenya; anyangaresi@gainhealth.org

**Keywords:** biofortification, biofortified crops, biofortified foods, biofortified food products, micronutrient concentrations, micronutrient deficiency, effectiveness, impact evaluation

## Abstract

Biofortification is the process of increasing the concentrations and/or bioavailability of micronutrients in staple crops and has the potential to mitigate micronutrient deficiencies globally. Efficacy trials have demonstrated benefits of consuming biofortified crops (BFCs); and in this paper, we report on the results of a systematic review of biofortified crops effectiveness in real-world settings. We synthesized the evidence on biofortified crops consumption through four Impact Pathways: (1) purchased directly; (2) in informal settings; (3) in formal settings; or (4) in farmer households, from their own production. Twenty-five studies, covering Impact Pathway 1 (five studies), Impact Pathway 2 (three), Impact Pathway 3 (three), Impact Pathway 4 (21) were included. The review found evidence of an improvement in micronutrient status via Impact Pathway 4 (mainly in terms of vitamin A from orange sweet potato) in controlled interventions that involved the creation of demand, the extension of agriculture and promotion of marketing. In summary, evidence supports that biofortified crops can be part of food systems interventions to reduce micronutrient deficiencies in farmer households; ongoing and future research will help fully inform their potential along the other three Impact Pathways for scaling up.

## 1. Introduction

Micronutrient deficiencies, defined as the lack of essential vitamins and minerals required in small amounts by the body for proper growth, development, and function, are widely prevalent globally and continue to be responsible for extensive morbidity and mortality [1]. Deficiencies in iron, vitamin A, zinc, iodine, and folate are the most common, with pregnant women and children under five years of age in low- and middle-income countries (LMICs) at the highest risk. Growing concerns about the widespread impacts of micronutrient deficiencies have been magnified by the poor nutritional quality of crops less resistant to drought and other stresses, which are likely to be exacerbated further by continuing climate change [2].

The burden of malnutrition, such as micronutrient deficiencies, is fueled by a food system that does not provide adequate amounts of the nutrients needed for much of the developing world. While the yield of staple crops has been greatly enhanced by the technologies of the Green Revolution, these same developments also promoted agricultural monoculture that reduced crop and, consequently, nutrient diversity [3]. Agriculture-based nutrition-sensitive interventions, such as biofortification of staple crops, can and perhaps should be designed as a sustainable and affordable means of improving crop nutritional quality, and subsequently the health of vulnerable populations [4]. Many LMICs with a high prevalence of micronutrient deficiencies rely on staple carbohydrates and energy-rich crops such as wheat, maize, rice, sweet potato, pearl millet, lentils, beans, and cassava that are affordable yet do not provide an adequate array of micronutrients to meet human needs, particularly when the food system transforms staples into ultra-processed foods [5].

Biofortification, the process of increasing the concentrations and bioavailability of essential nutrients in a staple crop through traditional plant breeding, agronomic practices, and/or genetic engineering, is a potential approach to combat micronutrient deficiencies at the population level. Crops can also be selected for resistance to stress, critical for extreme climate fluctuations [6]. Although breeding varieties that will meet future environmental challenges is already part of many national agriculture sectors, strengthening the pipeline of developing climate-smart crops with target nutrient levels remains a strategic priority [4]. HarvestPlus, a global organization dedicated to improving nutrition through crop biofortification, has tested and/or released hundreds of biofortified varieties of 12 types of crops in 128 countries [7].

A major strength of biofortification is that it can be self-sustaining and requires minimal future investment: Once a nutrient rich variety is developed and spread, only monitoring and maintenance expenditures are required. Biofortification is also potentially advantageous in sustainability and reach compared to other micronutrient interventions, such as supplementation and industrial (or large-scale) post-harvest food fortification, both of which require specialized infrastructure and may not be feasible in LMICs [8]. As an institutional means of combating nutritional deficiency, biofortification can be incorporated into existing food systems and agricultural practices, with observable effects in rural communities as agricultural households and communities consume their crops as well as urban consumers.

Effectiveness studies and surveys on biofortified crops aim to provide evidence for the concept of biofortification as an effective and cost-effective population-level nutrition intervention, as well as examine the active use and integration of biofortified crops in a community and subsequent changes in micronutrient status. As shown by controlled efficacy studies [4], biofortification improves micronutrient deficiencies at the population level. However, the effectiveness of biofortification across fundamental Impact Pathways—i.e., the pathways leading to particular contexts, in which biofortified foods and food products are obtained and consumed—in community settings needs to be examined to inform areas where future research is needed, as well as scale-up [7].

**Figure 1:**
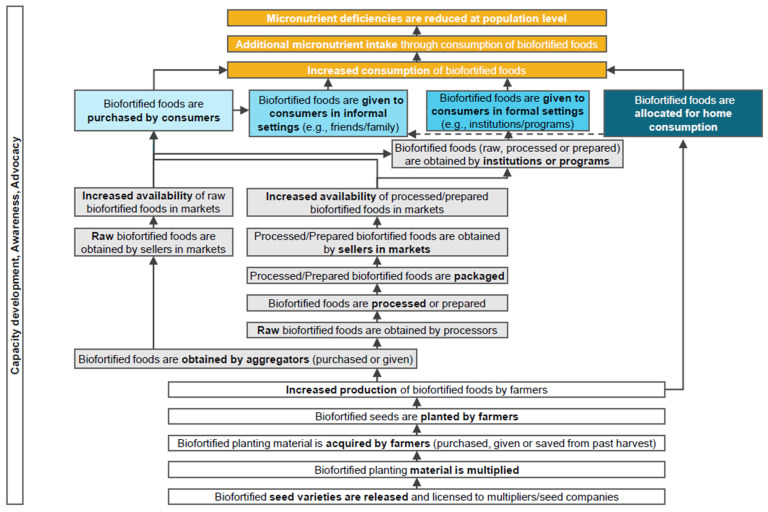
Biofortification Program Impact Pathway (PIP) framework [9]. Biofortified crop consumption may improve micronutrient deficiency via four Impact Pathways, highlighted by the blue boxes.

To our knowledge, no systematic review of biofortified crop(s) effectiveness studies and surveys in uncontrolled real-world settings has been conducted. Based on a biofortification Program Impact Pathway (PIP) (Figure 1), our objective was to summarize the evidence on the impact of the consumption of biofortified crops and food products in the four key pathways, through which biofortified foods are consumed. To the best of our knowledge, this conceptual framework is the only biofortified food procurement theory of change PIP diagram available.

The purpose of this review is to synthesize the current evidence for the effectiveness of consuming biofortified foods and food products to reduce micronutrient deficiencies at the population level, when biofortified crops are:

1. purchased directly by consumers;
2. given to consumers in an informal setting;
3. given to consumers in a formal setting; or
4. allocated by farmers for home consumption.

## 2. Materials and Methods

We registered the protocol for this review on PROSPERO (ID# CRD42021254461), the international prospective register of systematic reviews of the University of York and the National Institute for Health Research, on 11 June 2021 [10].

### 2.1. Inclusion and Exclusion Criteria

Any human population was considered eligible for inclusion.

We included studies utilizing micronutrient-biofortified crops-based foods and food products, including those that have undergone processing or cooking postharvest, that have been delivered or allocated in the case of own production as crops only or in the form of food products (as defined by trialists or study authors). Crops included those biofortified by conventional plant breeding approaches, consumed raw or in a cooked or processed form.

For Impact Pathway 1, we included studies that described the purchase and consumption of biofortified crops in terms of the frequency of purchase of a household, the proportion of households purchasing, the amount of purchased crops consumed, or the change in micronutrient status or other functional outcomes from purchasing and consuming biofortified crops.

For Impact Pathway 2, we included studies that described the consumption of biofortified crops through indirect means (neighbor-to-neighbor dissemination, gift, or payment in-kind). This was measured by the household’s frequency of purchasing crops indirectly, the proportion of households that acquire crops indirectly, the amount of crops purchased indirectly, or the change in micronutrient status or other functional outcomes as a result of the consumption of biofortified crops acquired indirectly.

For Impact Pathway 3, we included studies that described the consumption of biofortified crops in formal settings (for example, school meal programs, public distribution systems, hospitals) in terms of the frequency of consumption or amount consumed, the proportion of these formal settings including biofortified crops on the menu, or the change in micronutrient status or other functional outcomes as a result of consuming biofortified crops.

For Impact Pathway 4, we included studies, including controlled effectiveness studies and impact assessments/evaluations, that described the farmer’s household consumption of biofortified crops from the farmer’s household’s own harvest in terms of the frequency of consumption or amount consumed by the household, or the change in micronutrient status or other functional outcomes as a result of consuming their own harvest.

For our investigation of consuming or reusing parts of the plant that are not the primary target of biofortification, such as leaves, roots, or stems of a given biofortified crop, we included both studies conducted in populations that discuss consumption of these parts of the crop, as well as laboratory studies measuring micronutrient content in these parts of the crop.

We did not include interventions using agronomic biofortification methods, genetic engineering-based biofortification methods, or animal-based biofortified foods such as dairy products or meat from animals that consumed biofortified feed. These criteria were applied to the four Impact Pathways and the investigation of the consumption or reuse of parts of the plant that are not the primary target of biofortification, such as the leaves, roots, or stems of a given biofortified crop. We also excluded studies that examined quality protein maize (i.e., ‘QPM’), as our primary focus was on micronutrient biofortification.

Comparators, if used, included either (A) a non-biofortified (i.e., conventional) version of the same crop or food product; or (B) crops and food products fortified with the same micronutrient.

From our review of the literature, we reported the following outcomes and information reported for each Impact Pathway:

#### 2.1.1. Impact Pathway 1, Direct Purchase and Consumption

**Primary outcomes:**

1. The frequency of consumption or the amount of biofortified crops consumed obtained through direct purchase;
2. Changes in micronutrient status or prevalence of deficiency compared to consumption of purchased biofortified crops.

**Secondary outcomes:**

1. The proportion and/or frequency of households purchasing biofortified crops for consumption;
2. The amount of said crops purchased.

#### 2.1.2. Impact Pathway 2, Indirect Consumption

**Primary outcomes:**

1. The frequency of consumption or the amount of biofortified crops consumed obtained by indirect means;
2. Changes in micronutrient status or prevalence of deficiency with respect to consumption of biofortified crops obtained indirectly.

**Secondary outcomes:**

1. The proportion and/or frequency of households or household members receiving biofortified crops for consumption as a gift or payment in kind, and the amount of crops received/consumed;
2. The proportion of households or household members giving biofortified crops to neighbors, their own children or other family members for consumption, and the amount of crops distributed/consumed.

#### 2.1.3. Impact Pathway 3, Formal Consumption

**Primary outcomes:**

1. The frequency of consumption or the amount of biofortified crops consumed in the context of a formal setting such as school meal programs, public distribution systems, hospitals, etc.;
2. changes in micronutrient status or deficiency prevalence with respect to consumption of biofortified crops in the context of said formal setting.

**Secondary outcomes:**

1. The proportion and/or frequency of school meal programs, public distribution systems, or hospitals, etc., including biofortified crops on their rotation/menu.

#### 2.1.4. Impact Pathway 4, Farmer Household Consumption

**Primary outcomes:**

1. The frequency or amount of biofortified crops consumed by farmer households, as a portion of crops grown by the said household;
2. Changes in micronutrient status or prevalence of deficiency with respect to consumption of biofortified crops by farmers’ households, as a portion of crops grown by the said household.

**Secondary outcomes:**

1. Reasons why farmers retained a given biofortified crop for consumption;
2. Proportion of households retaining biofortified crops for home consumption;
3. Farmer household consumption of crops as predictors of other outcomes e.g., diarrhea;
4. Potential factors impacting dietary intakes of micronutrients versus biofortified crops.

We did not restrict any type of study design; we included randomized controlled effectiveness trials, case or country reports, program evaluations, etc. We only included studies that reported data from trials, laboratory experiments, and population surveys that tested crops that were indicated to be conventional or traditionally biofortified cultivars. Studies that modeled, predicted, or estimated how biofortified crop consumption could potentially impact our outcomes of interest were excluded.

In this review, we define the ‘study’ as the main report of a given trial; a study can include more than one publication or report if the trial is reported in additional secondary analyses. We define the ‘record’ as the specific citation, which may be a secondary analysis on the same trial population.

### 2.2. Literature Search and Methodology

We originally aimed to conduct a set of four reviews on biofortification and thus designed our search strategy to accommodate the topics (consumption of biofortified crops along the four Impact Pathways; acceptability and adoption; bioavailability, bioaccessibility, micronutrient retention; and efficacy trials) examined by all four reviews (see original protocol [10]).

We performed a search of relevant literature databases, including: MEDLINE (PubMed), AGRICOLA, AgEcon, CABI Abstracts (Web of Science) and organizational websites (e.g., Harvest Plus, CGIAR and partners).

As a preliminary assessment of the literature on biofortification, we conducted a broad search on MEDLINE (PubMed) on March 2021, using the following key terms: Biofortification(MeSH) OR biofortif*(tiab) OR “bio-fortif*“(tiab). This resulted in 1434 (not de-duplicated) results. After screening these results and ascertaining key words to use to increase the sensitivity of the search, we performed searches in additional databases, using broad or narrower searching depending on the topic focus of the database. These, including the original MEDLINE search, are summarized in Table 1:

We also manually searched organization websites and added additional unique articles to the screening pool. The results are included in Table 2.

We also identified 1146 potential citations outside of the original search during the screening process. These included studies that were: cited in review papers, yet did not include any variations in the term “biofortification” in their abstracts; not indexed in any of the literature databases described above and were thus missed by the original search; published recently in 2021, which we identified from journals’ table of contents alert feeds. Some of the latter included full-text versions of conference abstracts that were found and included in the original screening pool.

### 2.3. Data Screening and Extraction

The following actions were taken by authors S.L.H., J.T.K., N.H.M., E.M.K., and A.B:

S.L.H., N.H.M., E.M.K., and A.B. screened all records for eligibility, first at the title/abstract level and subsequently at the full text screening level.

S.L.H., J.T.K., N.H.M., E.M.K., and A.B. used a subset of articles to improve consistency among review authors.

S.L.H., N.H.M., E.M.K., and A.B. extracted data for each identified study, depending on its applicability to one of our two Aims, according to the following: study level details including authors or research group, study year, and location; study type such as efficacy, effectiveness, bioavailability/retention; method details including randomization scheme, population, how crops were biofortified; outcomes of the study.

### 2.4. Data Synthesis and Analysis

We conducted a narrative synthesis of findings from the literature, given the qualitative nature of information, as well as the variety of different outcomes. We have organized these by Impact Pathway.

## 3. Results and Discussion

**Figure 2:**
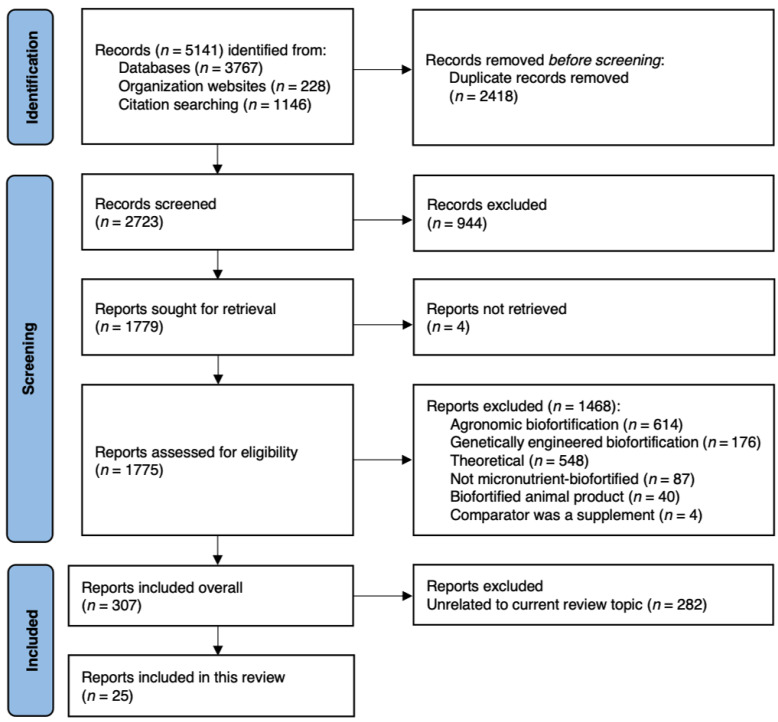
PRISMA Diagram [11].

For the four review topics, we found a total of 5141 records (Figure 2). Ultimately, we found overall 307 eligible records across the four review topics outlined previously. For the current review, we identified 25 studies in total, including the following numbers of studies per Impact Pathway (some studies involved multiple pathways):

- Impact Pathway 1, Direct Purchase and Consumption: 5
- Impact Pathway 2, Indirect Consumption: 3
- Impact Pathway 3, Formal Consumption: 3
- Impact Pathway 4, Farmer Household Consumption: 21

### 3.1. Impact Pathway 1, Direct Purchase and Consumption

We identified five studies that discussed the purchase and intended or actual consumption of biofortified crops (Table 3). Two studies were carried out in Rwandan populations [12,13], while we identified a study each from Mozambique [14], Nigeria [15], and Uganda [16].

In Rwanda, high iron bean consumption was examined in nearly 1400 bean farmers’ households nationally and locally in the Kigali, Southern, Western, Northern and Eastern provinces [12]. The percentage of households that consumed high iron beans in the past seven days was highest in Kigali (20%) and ranged from 8% to 14% in the other provinces and 9% nationally. Per capita, households consumed 197 g, 270 g, 204 g, 210 g, and 187 g daily in Kigali, southern, western, northern, and eastern provinces, respectively; nationally, households consumed 211 g daily. Although the data did not directly categorize the consumption of high-iron beans with a market source/purchase, the authors note that 2% of agricultural households did not grow high-iron beans, yet reported consuming them in the past seven days, indicating that households purchased high-iron bean grain from the market or received high iron beans as a gift or in kind payment [12]. Furthermore, 24% of households sold a portion of high iron bean grain from their first growing season, increasing to 26% within the subsequent growing season. This may be attributed to purchase and consumption by other households.

In Musanze district in Northern Rwanda, researchers surveyed adult consumers from 250 households who had previously been exposed to programs delivering biofortified planting material [13]. Of the whole sample, 15% (*n* = 37) of households reported ever consuming high-iron beans, while 10% (*n* = 21) reported current consumption of high-iron beans. One quarter of households (*n* = 57) knew where to buy or obtain high-iron beans, including a market/street stand (*n* = 43, 75%), the farmgate (*n* = 17, 30%), the store (*n* = 14, 25%) (*n* = 14, 25%), and/or the moving street vendor (*n* = 2, 3.5%). The same study also examined orange sweet potato (OSP) household coverage, finding that 10% of households ever consumed OSP while only 2% currently consume OSP. Only 11% of households (*n* = 26) reported yes to OSP availability. Where OSP was available, households reported being able to buy or obtain OSP only from the market/street stand (*n* = 19, 73%) and/or the farmgate (*n* = 11, 42%). No differences were observed between periurban (*n* = 25) compared to rural (*n* = 32) areas for high iron beans or OSP. The study authors determined that a lack of awareness and availability marked the primary barriers to the acquisition and consumption of high-iron beans and OSP.

In Maputo, Mozambique, one survey among consumers (*n* = 656) of OSP [defined as: (a) producer-household members; (b) producer-community members; (c) urban residents shopping at wholesale markets; (d) urban residents shopping retail markets or street corners; (e) urban resident buying from specialized OSP producers; and (f) urban residents buying at supermarkets] found that, on average, OSP is consumed about once in two weeks [14]. However, stratification among various groups of consumers, as previously defined, was not included. The survey results showed an average of 114 meals with OSP eaten per year and further classified the frequency of OSP consumption: Less than once per month (30%), less than once per week (65%), every day (25%). This resulted in an annual urban consumption of 14,000 tons (assuming a conservative mean consumption of 0.1 kg/person/meal) in Maputo. Subtracting 5000 to 6000 tons consumed from consumers’ own production, this translates to approximately 8000 to 9000 tons sold on the market and thus directly purchased for consumption.

In seven states (Akwa-Ibom, Benue, Enugu, Kwara, Kaduna, Osun and Taraba) in Nigeria, *n* = 735 consumers were surveyed about the consumption of biofortified crops [15]. Fewer than 3% of respondents indicated consuming biofortified OSP, cassava, or maize; biofortified food crops were only consumed occasionally, mostly depending on “whether the consumer came across them while shopping” [15]. Among those who bought biofortified food crops, OSP and cassava were consumed at similar frequencies among *n* = 95–113 respondents: occasionally (58–60%), weekly (22–26%), monthly (3.2–3.5%) or when in season (10.6–15.9%). The frequency distributions for maize strongly favored occasional consumption, with an occasional consumption of 71%, weekly 8.4%, monthly 3.3%, and in-season consumption of 17.3% (statistical significance not tested).

In Kampala, Uganda, 122 interviews with consumers in three markets revealed that 52% of those surveyed had purchased OSP for home consumption at least once [16]. Of the pool surveyed, 12% reported one-time purchases of OSP, 24% were repeat purchasers, and 17% were consumers who had inquired about and purchased OSP. The remaining included those unaware of OSP (22%) and those who had not purchased OSP (26%). Consumption or intake data for OSP was not reported among those who purchased OSP specifically, however data on customer perception of OSP, including taste, texture, and cooking attributes, were noted. Several barriers to the purchase of OSP were identified: perceptions of supply problems, unavailability, and the belief that sellers substitute regular sweet potatoes for OSP without consumer knowledge amplified by visual ambiguity. Physically, the primary negative perception was that OSPs are soft and mushy.

In summary, five studies were identified between 2010 and 2019 related to Impact Pathway 1. These studies were heterogeneous in location, design, methods, and outcomes and ranged from survey data to impact assessments. Purchase and consumption data were more specific for studies in urban areas, where frequency of individual purchase and consumption as well as annual city-wide consumption were reported [14,16]. The annual amount of biofortified crops consumed by a region or city in absolute or relative terms may be a useful index for assessing the uptake of biofortified crops by area. In contrast, in studies carried out in rural areas, purchases were mentioned in passing without consumption data, or crop consumption was reported, although it was not clear from where the crops were sourced [12,13,15]. However, this contrast between urban and rural is logical, given that urban residents may be more likely to have direct purchase of crops as their only source of biofortified crops, while in rural areas acquiring crops may come from multiple sources (see Impact Pathway 4).

As such, several gaps remain for Impact Pathway 1. Specifically, none of the five studies examined changes in micronutrient status as a result of consuming biofortified crops that were purchased or from any source of biofortified crops. Geographic breadth defines both a gap and a topic for further consideration. Just one study in each of only five countries in Africa reported on this specific Impact Pathway, leaving a dearth of data on the purchase and consumption of biofortified crops among similar populations in these countries, other countries in Africa, and South Asia and Latin America as a whole. However, having only one study reporting on this Impact Pathway per each of the countries mentioned could indicate that biofortified crops have not yet reached a stage of commercialization such that substantial volumes are available for direct purchases by consumers. Furthermore, only OSP, beans, and cassava were examined by studies. The direct purchase, consumption, and change in micronutrient status of pearl millet, wheat, rice, cowpea, or other crops targeted for biofortification remains unclear. More studies are needed to fully assess the potential reach and impact of the consumption of biofortified crops from direct purchases and, subsequently, the effect on micronutrient status, which may only be directly ascertainable from urban populations.

### 3.2. Impact Pathway 2, Indirect Consumption

We identified three studies that discussed indirect consumption of biofortified crops, such as neighbor-to-neighbor dissemination and consumption (Table 4). These included one study each from Mozambique [17], Rwanda [12], and Uganda [16].

In Zambezia, Mozambique, women and young children from 36 villages in Maputo were surveyed to determine if the Reaching End Users project, conducted from 2006–2009, had lasting effects on OSP and, subsequently, vitamin A intakes [17]. Nearly half of ‘treated’ households that received OSP vines and agricultural/nutrition extension and grew OSP reported giving some of their crop to neighbors; authors conclude that within-village exchange is likely an important source of OSP for OSP-consuming households.

Details on the Rwanda study [12] are described in Impact Pathway 1 above. In summary, among 1400 households in Rwanda, specifically in the Kigali, South, West, North, and East provinces, 2% of the farming households who did not grow high-iron beans reported consuming high-iron beans, indicating that the beans were received as a gift or in-kind payment, or possibly purchased from the market.

Details on the Uganda study are described in Impact Pathway 1 above [16], with additional details relevant to Impact Pathway 2 as follows. Briefly, most of the respondents in the 122 interviews who had served OSP had served them to their own child/children (84%) and spouses (79%). Some had served OSPs to other children (6%) or adults (14%).

To conclude, there were again very few studies describing Impact Pathway 2, and all were conducted in Africa. The remaining gaps are similar to those identified for Impact Pathway 1. Examining this pathway across more crop types and populations is needed, though increasing the geographical range, as well as surveying changes in micronutrient status as a result of consuming these crops indirectly.

### 3.3. Impact Pathway 3, Formal Consumption

We identified three studies that discussed the consumption of biofortified crops in formal settings (Table 5). These included a study from Nigeria [18], a study from Brazil [19], and a global survey of school lunch programs in 85 countries [20].

A study on the consumption of biofortified crops in formal settings was related to school meal programs. In Nigeria, schoolchildren aged 7–12 years (*n* = 556) participated in a four-week intervention and field experiment in 12 primary schools in Osun state [18]. In this study, OSP was introduced on five occasions as a complement to the existing school meal. The average proportion of OSP in meals compared to the total amount of school meal eaten was 58% ± 22% SD during the four-week intervention. Vitamin A status was not quantified.

Another study in the Itaguaí municipality of Rio de Janeiro, Brazil, described the consumption of several biofortified crops introduced in school meals among 5–12-year-old students enrolled in rural public schools [19]. It was not clear whether the school had incorporated biofortified crops into the regular menu or if the inclusion of these foods was on a trial basis for the purpose of the study. Biofortified OSP and biofortified cassava were served in 20-g portions, biofortified maize was milled into corn cake and served in 30-g portions, and biofortified beans were served in 50-g portions. A 20-g portion of biofortified OSP contributed 1816 µg total carotenoids and 1648 µg beta carotene, translating to 137 retinol activity equivalents (RAE) per 20-g portion (34.4% and 22.9% of the recommended dietary allowance (RDA) for children 4–8 and 9–13 years old, respectively). A 20-g portion of biofortified cassava contributed 162 µg total carotenoids and 143 µg beta carotene, translating to 11.9 RAE (3% and 2% of the RDA for children 4–8 and 9–13 years old, respectively). A 30-g portion of biofortified corn cake contributed 738 µg total carotenoids and 67 µg beta carotene, translating to 5.6 RAE (1.4% and 0.9% of the RDA for children 4–8 and 9–13 years old, respectively). Finally, 50 g of biofortified beans contributed 1.8 mg of ferritin and 0.79 mg of zinc. For ferritin, this translates to 10.8% and 13.5% of the RDA and for zinc, 15.8% and 9.8% of the RDA, for children aged 4–8 and 9–13-years, respectively. Only biofortified beans were compared with conventional versions, which contributed 0.75 mg of ferritin and 0.38 mg of zinc per 50 g serving, which equates to 7.5–9.4% of the RDA and 4.7–7.6% of the RDA, respectively. In this study, serum biomarkers for vitamin A, iron, or zinc were not examined.

We did not identify any other studies that examined the consumption of biofortified crops among schoolchildren in a formal setting such as a school meal program. This finding is paralleled by a comprehensive report on school meal programs worldwide [20], which found that in 70 countries reporting from the school years 2017–2018 or 2018–2019, only 12% of school lunch programs in 11 countries used biofortified crops. To explain this lower prevalence, the authors of this report note that biofortification is a relatively new option that is not yet widely available, compared to the inclusion of industrially fortified foods (68% of programs) and micronutrient supplements (22%). As a result, the report highlights some pressing issues and questions:

“Are those responsible for the implementation of school meal programs knowledgeable about fortification and biofortification options and benefits? What kind of cross-sectoral collaboration is required if fortification programs are to be initiated or scaled up? Where are some fortification success stories, and what can be learned and shared from those experiences?” [20].

An updated global report was planned for 2021, however, it has not yet been published at the time of writing this review, which may help answer some of these questions.

We identified only two primary studies that examined biofortified crop consumption in a formal setting, a controlled study using an existing school lunch program. We did not find any studies describing school lunch programs that already include biofortified crops on their rotating menus, which was reflected in the global survey of school meal programs conducted in 2019. We also did not find studies that examined biofortified food consumption in the context of other formal settings, such as public distribution systems or other institutions, such as hospitals. Given that biofortification is a relatively new nutrition intervention, we anticipate that more studies will be found for this Impact Pathway in the future and emphasize the need to conduct more studies on this pathway.

### 3.4. Impact Pathway 4, Farmer Household Consumption

We identified 21 studies related to the consumption of biofortified crops by farmers’ households that grow that crop (Table 6).

To date, biofortification has been implemented in a few selected countries specifically targeting this Impact Pathway; as a result, more data were found for this Impact Pathway compared to Impact Pathways 1–3. The identified studies were often analyses conducted in the context of a larger survey, program or project, such as the Reaching End Users (REU) Project, the Rwanda High Iron Bean Survey, the Reaching Agents of Change (RAC) Project, the Building Nutritious Food Baskets Project (BNFB) and the Sweetpotato Action for Security and Health in Africa (SASHA) Project. These are introduced below. Additionally, we discuss several studies that were conducted outside of the context of a larger program.

#### 3.4.1. Reaching End Users (REU) Project

The objective of the REU Project was to introduce OSP cultivation at home, assess adoption OSP, and determine whether adopting and consuming OSP resulted in improved vitamin A intakes among populations at risk for vitamin A deficiency, such as farm households in Uganda and Mozambique [22,23,42].

The REU project was modeled on a previous smaller-scale (*n* = 741), quasi-experimental effectiveness study carried out between 2003 and 2005 [21], which introduced OSP to rural communities in Zambézia province, a resource-poor and drought-prone area of central Mozambique. This controlled intervention ultimately reduced the prevalence of vitamin A deficiency in young children by creating a 2-year intervention that simultaneously linked and promoted the following: (1) farmer access to OSP vines, including farmer extension; (2) nutrition knowledge and demand creation for OSP; and (3) market development to ensure sustainability. Activities included community theater, radio spots, and visibility at local markets. An attempt to integrate farmer extension covering various agricultural topics and nutritional education on infant/young child feeding and hygiene practices was also incorporated by having participants attend 9 to 12 sessions over a 1-year period. The control group, on the other hand, was exempt from participation in all extension and educational activities. Given the high prevalence of vitamin A deficiency in this area, this study provided all children, in the intervention and control groups, vitamin A capsules (60,000 µg retinyl palmitate per capsule); These capsules were administered after blood sample collection at three time points (baseline, midpoint, and endpoint).

At the end of the 2003–2005 study (*n* = 741), the food frequency questionnaires revealed that OSP consumption increased to a greater extent in the intervention area compared to the increase in the control area; 50% of the children in the intervention area ate OSP in at least three of the last seven days, compared to less than 10% of the children in the control group [21]. The prevalence of vitamin A deficiency among non-breastfed children was 10% lower in the intervention group compared to the control group. Furthermore, the prevalence of vitamin A deficiency in healthy children (those with low or no inflammation) in the intervention group decreased from 60% to 38%, compared to the absence of change in prevalence in the control group. Furthermore, the mean unadjusted serum retinol in intervention children was 0.07 µmol/L higher (mean: 0.74 µmol/L) than in control children (mean: 0.67 µmol/L). The adjusted mean serum retinol concentration did not differ between the groups at baseline and did not change in the control children, yet increased by 0.10 µmol/L in the intervention children. As highlighted by various statistical measures, the intervention group provided multiple instances of improved nutritional status and adoption.

Based on the design and success of the Mozambique intervention in 2003–2005 [21], the Reaching End Users project was adapted and expanded for both Mozambique and Uganda. The REU project distributed the OSP to 10,000 farm households in Uganda and 12,000 households in Mozambique randomized to a more intensive Model One intervention (i.e., ‘intensive’) or less intensive, cheaper (by about 30%) Model Two (i.e., “reduced”, “moderate”, or “less intensive”) intervention. The REU intervention had three components: seed systems, in which OSP vines were distributed to households and farmers received training on how to grow OSP; demand creation, where mothers were offered training and education on the benefits of consuming OSP, how to cook OSP, and other health messages; and marketing, which aimed to increase visibility and demand for OSP in local markets. Intense Model One included all three components throughout the 2-year project duration; the project; Reduced Model Two included vine distribution, however with no training in Year 2 to minimize costs [25]. This study also included a control arm that did not receive interventions.

Several analyses conducted on data from the REU project revolved around consumption by farmer households who adopted OSP cultivation after participating in one of the trial arms. The impact of the REU interventions on mean vitamin A intakes (µg Retinol Activity Equivalents, RAE/day) among target populations: children 6–35 months (Mozambique), 3.5–6 years (Mozambique), 5–7 years (Uganda), and women 15–49 years (Mozambique and Uganda) were analyzed [22,23,26]. Across both OSP adopters and nonadopters in the intervention groups, total vitamin A intakes increased significantly as a result of increased OSP intake (in boiled form) in the three target populations in Mozambique and Uganda, with no significant differences between intensive Model 1 and reduced Model 2 [22]. A causal mediation analysis found that the added knowledge of nutrition messages minimally affected adoption and subsequent intake of OSP, and increased vitamin A intakes were largely explained by adoption itself and not nutrition knowledge gained [25]. However, specifically in Uganda, a large share of impacts on vitamin A intake could not be explained by mediating variables such as the contribution of nutritional knowledge to the adoption decision [25]. Overall, given the similarity of the results between Models 1 and 2 and the mediation analysis, the authors conclude that similar impacts could be achieved even with a reduced number of nutrition trainings.

In Mozambique, OSP accounted for 47–60% of all sweet potatoes consumed in the Model 1 and Model 2 groups in the age groups, indicating a moderately high degree of substitution, compared to the control group where 20–24% of all sweet potatoes consumed were OSP [26]. Furthermore, vitamin A intake doubled for all three age/sex groups by the endline, compared to a baseline RAE intake per day below the EAR standard (see Box 1). OSP was the dominant source of vitamin A in the diet, providing 71–84% of total vitamin A in all groups. Furthermore, there was a significant net reduction in the prevalence of low vitamin A intake in the intervention groups compared to the control.

Box 1 **Vitamin A intake estimated average requirements for women and children.**

For reference, the estimated average requirement (EAR) for vitamin A intake for children aged 12–35 months is 210 µg RAE/day, for children aged 4–8 years is 275 µg RAE/day, and for women it is 500 µg RAE/day, according to the Institute of Medicine [43]. These recommendations may vary by country guidelines.

In Uganda, where baseline vitamin A intake was approximately equal to EAR, vitamin A intake increased by two-thirds for younger and older children and nearly doubled for women as a result of OSP consumption [22]. At follow-up, the proportion of all sweet potato consumed as OSP was ≥31–38% in the intervention groups, compared to ≤4% in the control group [27]. Considering only households that adopted OSP cultivation, vitamin A intake was approximately 30% higher in both countries. In Mozambique, own-produced OSP can be expected to provide these levels of intakes for 2–3 months out of the year, and in Uganda at 4–5 months per year. Overall, OSP contributed to 78% of total vitamin A intake in Mozambique and 53% in Uganda [23].

Furthermore, the impact of vitamin A intake from OSP on serum retinol was assessed among children 3–5 years old with inflammation-adjusted serum retinol <1.05 µmol/L at baseline, comparing the intensive group to control [27]. Among a subset of children with complete data on serum retinol and serum inflammatory markers (*n* = 472), OSP vitamin A intake did not affect the prevalence of vitamin A deficiency (defined as serum retinol <0.70 µmol/L), however the authors observed a non-statistically significant 7.6 percentage point reduction in the prevalence of vitamin A insufficiency (serum retinol <1.05 µmol/L) in children (*p* = 0.09). In children with complete data (*n* = 396) on important covariates (age, deworming status and vitamin A supplement intake) the impact of OSP-sourced vitamin A intake was associated with a 9.5 percentage point reduction in the prevalence of vitamin A insufficiency (*p* < 0.05) relative to the control. In women, the sample size was limited, as only 95 women had serum retinol adjusted for inflammation <1.05 µmol/L at baseline. No impact of intervention was observed compared to the control in this group.

The authors ran a similar second analysis in Uganda that only accounted for follow-up data [27]. In children with baseline inflammation-adjusted serum retinol <1.05 µmol/L and dietary intake data (*n* = 199), vitamin A intake from OSP was positively associated with serum retinol and with lower prevalence of vitamin A insufficiency and deficiency. For women with baseline inflammation-adjusted serum retinol <1.05 µmol/L and dietary intake data (*n* = 33), vitamin A intake was only associated with a lower prevalence of vitamin A insufficiency. Serum retinol analyses were not performed in Mozambique in the context of the REU project; the authors stated that this was due to the smaller-scale, earlier study having already shown a positive effect of OSP on serum retinol in the target population examined in the REU project [26].

In 2012, a three-year follow-up evaluation of the REU project was performed [17]. Dietary intakes among women of reproductive age and children were collected in the same population since the endpoint. Vitamin A intake remained higher in the treated villages compared to the control villages even among children under 3 years of age, who had not yet been born during the original intervention. Among all children 6–59 months of age (*n* = 262) and all children 6–35 months of age (*n* = 153), the intervention group had a higher frequency of OSP consumption. In terms of vitamin A intake among children, the average effect of the REU intervention was between 103.5–111.1 µg RAE, representative of approximately half of the US RDA of 210 µg RAE per day, compared to the control group that did not receive intervention. In mothers, the average effect of intervention on vitamin A intake was around 280 µg RAE. Further analysis revealed that the total difference in vitamin A intake in the treated compared to control groups was almost entirely due to OSP intake. Despite this, vitamin A status was not assessed at the three-year follow-up. In 2011, unfortunate weather conditions resulted in many households losing their vines. The authors conjecture that the long-term impacts of the project would have been amplified given the improved retention of the vines.

Another analysis examined OSP consumption as a predictor of diarrhea incidence [24]. In the Mozambique REU trial, *n* = 781 children under five years of age (1321 observations total) were analyzed at the endpoint. While consuming OSP was recorded in seven-day food frequency questionnaires, the actual OSP or vitamin A intakes were not reported in this ancillary analysis; OSP consumption did, however, reduce diarrhea duration and prevalence by 11.4 percentage points in all children under age five, and nearly 19 percentage points in children under age three.

In another ancillary study, the role of gender dimensions of intrahousehold bargaining power and decision making was examined in reference to the impact of women’s bargaining power on children’s dietary intakes of vitamin A [28,29]. This study was on households (*n* = 327) with complete bargaining variables (defined as women’s bargaining power based on control over land; over nonland assets, and interactions) wherein the woman has primary control over decision making and are more likely to have parcels of land containing OSP, and subsequently facilitate diffusion by sharing OSP vines, and, as such, there was no impact of women’s bargaining power on dietary intakes by children. The authors speculate that this may indicate that women were unable to use their bargaining power to increase their child’s vitamin A consumption or that husbands and wives have the same preferences about the nutritional status of children, rendering bargaining variables functionally insignificant.

Controlled effectiveness studies conducted in Uganda and Mozambique show evidence that an intervention including agriculture, demand creation/behavior change, and marketing was successful in (1) increasing OSP vitamin A consumption and (2) improving vitamin A status in vulnerable groups such as mothers and young children.

#### 3.4.2. Rwanda High-Iron Beans Survey

In Rwanda, a largely agricultural nation with two growing seasons (Season A: September–February, Season B: March–June), a high degree of iron deficiency [44] coupled with reliance on bean growth and consumption has provided an opportunity for improving iron status through high iron biofortified beans; such beans (10 varieties of high iron bush and climbing beans) were introduced to Rwanda in 2010. In 2015, an impact assessment study was conducted in the East, West, North, South, and Kigali areas of Rwanda during Season B to understand the reach of high-iron biofortified beans (released five years prior) to rural Rwandan bean farmers [12]. First, a listing survey was conducted at the beginning of Season B during the planting period, which enlisted 19,575 households in 120 randomly selected villages throughout the country (93% were producers of any types of climbing or bush beans). From this listing survey, 28% of bean farmers (equivalent to 500,000 households) had grown at least one high-iron bean variety in at least one season between 2010 and 2015 [30]. In Season B of 2015 specifically, 20% of bean farmers (350,000 households) reported growing a high-iron bean.

Immediately after bean harvesting took place, a detailed household survey was conducted among 1400 bean-farming households, assessing the proportion of high-iron beans used for home consumption among other questions such as awareness of the crop, perceptions, land-use, and characteristics of adopters [12]. On average, 88% of households indicated that they kept a portion of their high iron bean harvest for home consumption in the first season they grew high iron beans, and 97% of households that continued to grow high iron beans kept a portion for home consumption. In total, the average percentage of harvest used for household consumption was 62% for high-iron bean varieties and 69% for non-biofortified beans.

Related to bean consumption (described in Impact Pathway 1 and repeated here), the percentage of households consuming high-iron beans in the past seven days was highest in Kigali (20%) and ranged from 8% to 14% in other provinces and 9% nationally [12]. Per capita, households consumed 197 g, 270 g, 204 g, 210 g, and 187 g daily in Kigali, southern, western, northern, and eastern provinces, respectively; nationally, households consumed 211 g daily. However, the source of these beans (from own harvest or via direct purchase) is not explicitly specified.

A secondary analysis investigated the effect of the most widely adopted high-iron bean cultivar, RWR2245, on bean consumption among 1400 households [32,33]. Over a 12-month period, growing RWR2245 for at least one of the two growing seasons increased the length of time beans were consumed from farmers’ own production by ~20 days. Furthermore, the average quantity of beans consumed monthly from self-production among adopters was ~4 ± 2.29 kg per adult male equivalent in *n* = 238 participants. However, it is unclear whether high iron and/or nonbiofortified beans were included in this monthly total. In another analysis of 1400 households, the importance of the International Center for Tropical Agriculture (CIAT) genebank for the development and adoption of seven iron-biofortified varieties of climbing beans introduced to Rwanda [31] was carried out to build on previous research using bush beans. Households growing iron-biofortified varieties consumed beans from their own production for 8.21 months on average and purchased beans (not indicated to be high-iron varieties) from the market for 3.4 months. In contrast, growers of local varieties consumed beans from their own production for 7.3 months and purchased them (again not indicated to be high-iron varieties) for 4.33 months. Adopting climbing varieties of beans did not have an impact on the consumption of high-iron beans by households. The reduction in market purchases of (conventional) beans may translate into increased consumption of high-iron beans among adopters.

In contrast to the REU project on OSP described earlier, the Rwanda project described above was not a controlled study and may be more representative of real-world uptake and consumption. Again, a follow-up survey that examines the household consumption patterns of farmers’ own production to assess sustainability would be fundamentally useful. Additionally, serum ferritin and other iron indicators were not used to determine the effect of consuming high-iron self-produced beans in this population, leaving a significant gap in the evidence for effectiveness of the crop in mitigating iron deficiency.

#### 3.4.3. Reaching Agents of Change (RAC) Project

The RAC project was a 3.5-year initiative (2011–2015) that advocated for policy change and increased investments, including advocacy toolkits to scale up OSP to combat vitamin A deficiency; the design and history of this project are described in a recent review [34]. In an ex-post survey of the RAC project, it was noted that production of OSP has remained on a largely small scale (land sizes 1 acre to 1.5 acres) and is predominantly used for home consumption [34]. However, the resulting effects of the study are ongoing and are expected to benefit at least 600,000 households directly, having reached 309,974 direct beneficiaries thus far in 2021. An updated assessment with quantification of OSP consumption from farmers’ own production is needed.

#### 3.4.4. Building Nutritious Food Baskets (BNFB) Project

BNFB was a three-year project (2015–2018) in Nigeria using a multi-biofortified crop approach to reduce micronutrient deficiencies and was based on lessons learned from the previous RAC project [15]. In Nigeria there is high prevalence of vitamin A and iron deficiencies, particularly in women and young children [45]. In this project, provitamin A maize, OSP, and yellow cassava were included in the adoption scale-up efforts. In a BNFB situation analysis, farmers (*n* = 420) were surveyed in the Benue, Kaduna, Akwa-Ibom, Taraba, and Osun regions. For most farmers, the cultivation of biofortified crops was mainly for local consumption: 61.1% cited this reason, compared to reasons such as ‘easy to cultivate and manage’ (0.9%), ‘available and environment conducive’ (1.6%), commercial and financial benefits (30.3%) or other (6.1%). However, household consumption data revealed that fewer than 3% of respondents consumed only biofortified sweet potato, cassava, or maize, compared to 81–91% of respondents who reported consumption of only conventional versions of these crops. A slightly higher proportion reported consuming both biofortified and nonbiofortified crops (8–15%). Serum retinol and other measures of vitamin A status were not reported.

#### 3.4.5. Sweetpotato Action for Security and Health in Africa (SASHA) Project and Marando Bora Project

The SASHA project explicitly integrated agriculture and nutrition interventions into antenatal care (ANC) and postnatal care (PNC) healthcare services in western Kenya [35]. The project targeted households with pregnant or lactating women, promoting improved nutrition education using inputs such as vouchers for OSP planting materials and enhanced agricultural extension [46]. Through increased uptake of health services and production and consumption of OSP, it was hypothesized that the SASHA project would improve the nutritional status of pregnant and lactating women.

The health facilities were selected and randomized to intervention and control groups; in the intervention facilities, women attending the ANC or early PNC clinics received enhanced nutrition education, including counseling, maternal and infant and young child feeding practice guidance, vitamin A information and its importance for maternal and child health, and OSP as a vitamin A rich food [35]. Women also received two vouchers for 100 OSP vine cuttings to redeem in secondary vine multipliers near health clinics, and women were linked with ‘clubs’ of pregnant and lactating women where community health workers conducted monthly sessions on nutrition and health, including foods rich in vitamin A and OSP. The control facilities also included these interventions, although they excluded clubs, agricultural and OSP activities.

In the intervention group, the proportion of women who consumed any OSP in the previous seven days increased from 8% at baseline to 28.7% at the end of pregnancy, 35% at the four-month postpartum visit and 55% at the nine-month postpartum visits [35]. On the contrary, one woman reported consuming OSP at any time point in the control group, preventing analysis impact estimates for the consumption of OSP. In a subsample of women (*n* = 206) who completed dietary recalls, the intervention was associated with significantly higher intakes of beta-carotene and RAEs at 8–10 months postpartum, as well as vitamin A adequacy defined as meeting the EAR or DRI. OSP also ranked higher in percentage contributions to beta-carotene and RAE intake among mothers in the intervention group compared to control mothers. There were no significant impacts of the intervention on mean concentrations of retinol binding protein (RBP) (or on hemoglobin) during follow-up. The intervention was also associated with 45% reduction in the odds of vitamin A deficiency (RBP < 1.17 µmol/L) at 9 months postpartum (OR: 0.55; 95% CI: 0.33, 0.92; *p* = 0.01).

Two studies analyzed data from the Marando Bora project [36,37]. The Marando Bora project, a component of the SASHA project, took place between 2010 and 2013 and was carried out in four regions of the lake zone of Tanzania; Mara, Mwanza, Shinyanga, and Kagera. The project, which aimed to increase the availability of OSP planting material for farmers at the start of the rainy season, promoted early planting and allowed farmers to increase root yield by taking advantage of unpredictable rainfall [36]. After identifying farmers and farmer groups who meet the eligibility criteria to become decentralized vine multipliers (DVMs), rapid multiplication techniques were taught [see [36]. A subsidized voucher system was used to distribute the OSP varieties from the DVMs. The voucher beneficiaries included those who had access to land, households with children under the age of five years and women-headed households; these beneficiaries received 200 cuttings of vines (planting material) in exchange for the voucher. Sensitization and promotional materials were also provided to farmers to raise awareness, including the nutrition benefits of OSP varieties. At the end of 2012, the Marando Bora project was estimated to have reached 110,000 farmers with vines [47]. The effect of the Marando Bora project on the proportion of roots of OSP from total sweet potato production for the household was estimated among *n* = 434 households at the end point: after exposure to the project, the proportion of OSP from total sweet potato production increased by 16 percentage points for participants’ households relative to nonparticipant households. The project did not quantify its effect on the frequency of consumption of OSP, and instead all vitamin A foods were categorized as variables. Vitamin A status was also not measured, although the authors note that this project did not influence nutrition results given the focus of the project on providing access to better quality vines and information on agronomic practices.

In the second analysis in Tanzania using Marando Project survey data [39], the authors noted that among *n* = 919 respondents, farmers ‘mainly grow (OSP) for home consumption, although some produce both for home consumption and sale”; factors related to the proportion of total OSP roots in total production for the household were analyzed. However, OSP consumption data, vitamin A intake, and vitamin A status were not quantified.

#### 3.4.6. Other Studies

In Maputo, Mozambique (see details of this study in Impact Pathway 1, some of which are repeated here), survey results showed that consumers eat OSP less than once a month (30%), less than once a week (65%) and every day (25%), with an average of 114 meals with OSP eaten per year, resulting in an annual urban consumption of 14,000 tons (assuming a conservative mean consumption of 0.1 kg/person/meal) [14]. The authors cross-checked these data with information from other sources, including unpublished 2015 IAI (*Inquérito Agrário Integrado*) data and Food and Agriculture Organization of the United Nations Statistical Databases (FAOSTAT) and Governo da Província de Manica (GPM) yield estimates, finding approximately 346 hectares of sweet potato inside Maputo City, equivalent to 5000–6000 tons coming from the consumers’ own fields for consumption. Vitamin A status indicators were not described.

The adoption and consumption of iron and zinc biofortified black beans, specifically the KK15 varietal, was briefly discussed in a secondary study among 48 farmer groups (20–50 members per group) of a randomized controlled trial in Kenya [38,39]. In the early period of KK15 adoption, while training sessions were being implemented, farmers planted only small areas with the new varietal to test the feasibility of adding this crop to their rotation; these small amounts were reported to have been ‘primarily consumed at home and not marketed’. The authors speculate that it is possible that the marketing training will have a larger effect on consumption patterns after increasing the area cultivated with KK15 at a later stage. Iron status indicators were not described.

The sweet potato value chain was examined in Odisha, India, to understand the potential of OSP to reduce vitamin A deficiency, a major public health problem [40]. Unlike countries in Africa, sweet potato is not a staple in Odisha, despite the state’s heavy sweet potato production. From the survey of *n* = 142 consumers, *n* = 310 producers and consumers, *n* = 25 aggregators, *n* = 12 wholesalers, and *n* = 25 retailers, it was found that sweet potatoes are consumed in mainly small amounts on auspicious occasions, with low awareness of the nutritional properties of OSP and of OSP itself. Only 11% of the farmers surveyed grew OSP. This study demonstrated the need to generate awareness and demand in this region.

We also examined a preliminary analysis of a controlled effectiveness study in eastern Guatemala in adolescent girls (unpublished [41]; personal communication from Dr. Erick Boy). This 2016–2019 study sought to assess the socioeconomic and nutritional impact of an intervention that delivered biofortified bean seed to farmers on bean production, the amount of beans saved for consumption, and the resulting iron intake. Households in 120 communities in seven municipalities in eastern Ghana were cluster randomized to receive high iron bean seed along with agronomic training and nutrition information, or to a control group. From 1764 households who had a positive harvest of beans, the intervention group saved a greater quantity of beans for consumption at harvest compared to the control group. Furthermore, the girls in the intervention group were shown to have consumed more iron per day and received more iron from the beans compared to the girls in the control group.

Blood biomarkers, including hemoglobin and serum ferritin, as well as the soluble transferrin receptor (sTfR) were also measured in 1214 participants (*n* = 754 in the intervention and *n* = 460 in the control group) [41]. The baseline prevalence of anemia, adjusted by age and altitude, was 1.8% and 1.3% in the intervention and control group, respectively, and 2.3% and 2.6%, respectively, at the endpoint. Baseline iron deficiency (serum ferritin <15 µg/L) was 4.9% and 4.6% in the intervention and control group, respectively, and 13.1 and 15.2% at the endpoint, respectively. Finally, the baseline iron deficiency measured by sTfR >8.3 mg/L was 3.4% and 3.0% in the intervention and control group, respectively, and then 2.8% and 3.5% at the endpoint, respectively. No significance testing was reported in these preliminary analyses. There was a small yet significant increase in hemoglobin (g/dL) between baseline and endpoint in the control group although not in the intervention group, and there were no significant differences in the changes in serum ferritin and sTfR between baseline and endpoint.

In summary, we identified 21 studies related to the Impact Pathway 4, making the impact of biofortified crop consumption from farmers’ own production the most reported of the Impact Pathways. The studies focused primarily on OSP, along with a smaller number of studies on biofortified common, bush, climbing, or black beans. Of the studies that examined micronutrient status, serum retinol or retinol-binding protein were measured in three analyses [21,27,35]. In general, interventions that combined demand creation, agriculture extension, and marketing were effective in increasing OSP consumption and reducing vitamin A deficiency (when measured).

Despite this, several gaps persist for this Impact Pathway. Measuring micronutrient status, including serum ferritin from iron-biofortified crops such as beans, as a result of consuming crops from one’s own production, requires adequate reporting to fully assess effectiveness and impact. The expansion of research on this Impact Pathway must also include a large diversity of crops, such as pearl millet, wheat, cassava, and cowpeas, from a greater diversity of geographies.

## 4. Conclusions

In this review, we examined the evidence on biofortified crop consumption and its impact on micronutrient status across four Impact Pathways: (1) biofortified crops purchased directly by consumers; (2) biofortified crops given to consumers in an informal setting such as neighbor-to-neighbor dissemination or gifts; (3) biofortified crops given to consumers in a formal setting such as a school lunch program; or (4) allocated by farmers growing the biofortified crop(s) for consumption by their household. We found very few studies for Impact Pathways 1, 2, and 3. Several studies reported data relevant to Impact Pathway 4. Impact Pathway 1 and Impact Pathway 2 had more detailed and specific data for populations in urban areas. In contrast, these Impact Pathways were more anecdotally discussed in rural populations. These findings make sense, as rural households can acquire biofortified crops from multiple sources (e.g., own production, neighbors, and purchases) compared to urban settings, where the market can be the only source of a biofortified crop and therefore more quantifiable. Only two reports were found for the Impact Pathway 3 and neither quantified the amount of biofortified crops consumed. Biofortified crop consumption in formal settings such as school meal programs, hospitals, or other institutions may be less common, and subsequently few studies have been performed on its effectiveness and impact. In contrast, biofortification has been rolled out in a few selected countries over the past three decades to investigate its contribution to Impact Pathway 4 and, as a result, several studies were identified. So far, evidence has found that OSP consumption improves vitamin A status in populations who adopt and grow sweet potato and allocate some harvest for their own consumption, through interventions involving demand creation, agriculture extension, and marketing.

Several gaps remain. To fully understand the potential of biofortification as a sustainable intervention and scale up its reach in the total population, it is important and necessary to move toward generating data on its Impact Pathways among other populations such as urban and nonfarmer rural, in addition, the expanding the knowledge on rural farmers in other settings such as South Asia and Latin America and in non-research settings (i.e., implementation of programs). For example, generating standards along the value chain for grain trade that are internationally applicable and tailored to the needs of grain traders will be critical for commercialization and expansion [48,49]. Such data will help us to understand whether biofortification is commercially viable and can exist independently of large programs and/or effectiveness studies. Biofortification needs to be mainstreamed by all relevant Consultative Group for International Agricultural Research (CGIAR) centers and National Agricultural Research System (NARS) (and hopefully private staple seed producers) to create enough supply of high yielding, climate smart, nutrient dense varieties/hybrids to capture a market share that is big enough to even expect to find a measurable degree of biological impact. Surveys that collect dietary intake where there are biofortified varieties available, on the other hand, should confirm the reported consumption/availability/access of biofortified materials by properly sampling these crops from fields and markets and measuring the micronutrient(s) contents. Relatedly, studies must measure and report changes in micronutrient status where feasible as a result of consuming biofortified crops in all four Impact Pathways to fully realize the goal of the program’s Impact Pathway (PIP) of reducing micronutrient deficiency; only a few effectiveness studies from Impact Pathway 4 have achieved this so far. However, it should be noted that these studies reported changes in micronutrient status from effectiveness studies conducted under largely controlled conditions such as voucher or vine distribution and education. These require follow-up analyses to determine if interventions and subsequent changes in micronutrient status were sustained and could be carried out without the context of the study. In summary, the evidence accumulated so far supports that an agricultural intervention like biofortification can be part of nutrition-direct and nutrition-smart food systems to reach Sustainable Development Goal #2, Zero Hunger, and more research is needed to fully understand its potential and reach scale globally.

## Figures and Tables

**Table 1:** Search strategy across included databases.

Database Name	Final Search String	Date of Search	Records (#)
MEDLINE	Biofortification(MeSH) OR biofortif*(tiab) OR “bio-fortif*“(tiab)	9 March 2021	1434
AgEcon	All of the words (biofortif*) in All Fields OR All of the words (bio-fortif*) in All fields	7 April 2021	73
AGRICOLA	TX (biofortif* OR bio-fortif*) AND TX (Adopt* OR Farmer* OR Household* OR Accept* OR Sensory OR DALY OR “disability adjusted life year*” OR Market* OR School meal program* OR Retention OR Mill* OR Process* OR Stor* OR Cook* OR Polish* OR Bioavailab* OR Cost-effectiveness OR Bioaccessib* OR Bioactiv* OR Efficacy)	7 April 2021	722
CAB Abstracts	TS = biofortif* OR TS = bio-fortif* AND TS = (Adopt* OR Farmer* OR Household* OR Accept* OR Sensory OR DALY OR “disability adjusted life year*” OR Market* OR School meal program* OR Retention OR Mill* OR Process* OR Stor* OR Cook* OR Polish* OR Bioavailab* OR Cost-effectiveness OR Bioaccessib* OR Bioactiv* OR Efficacy)	7 April 2021	1538
TOTAL			3767

*: Truncation symbol or wildcard syntax used in PubMed, to search for variant words or spellings. #: number of.

**Table 2:** Results from hand-searching organization websites.

Organization Website	Studies Identified on 7 April 2021
HarvestPlus	75 (manual)
CIMMYT Publications Repository	0 (captured in other databases)
IITA	2 (manual)
CIAT	0 (captured in other databases)
IRRI	0 (captured in other databases)
ICRISAT	151 = ”biofortif*”
ICARDA	0 (irrelevant)
TOTAL	228

*: Truncation symbol or wildcard syntax used in PubMed, to search for variant words or spellings.

**Table 3:** Characteristics of included studies for Impact Pathway 1, Direct Purchase and Consumption.

Country, Setting	Study Design	Population, *n*	Biofortified Crops Involved, Food Product or Processing	Outcomes Reported	Ref.
Rwanda, rural	Impact assessment study, including a listing survey and a detailed household survey in 2015 to examine the impact of releasing high-iron beans in 2010.	Farmer households from listing survey, *n* = 19,575 households in 120 randomly selected villages in beginning of season B in 2015; plus 1397 bean-farming households; Farmer households; *n* = 422	Iron bush beans released in 2010: RWR2445 RWR2154 MAC44 RWV1129 Year released: 2012 RWV3006 RWV3316 RWV2887 RWV3317 CAB2 MAC42	Frequency of consumption Consumption per capita	[12]
Rwanda, rural, peri-urban	Household surveys in 2019	Households, *n* = 250	Iron beans: cultivar NR PVA OSP: cultivar NR	Current or ever consumption Sources to purchase from	[13]
Mozambique, urban	Analysis using several data sources including government statistical data, primary data on prices of sweet potato roots, semi-structured interviews, and a survey among Maputo City residents regarding production and consumption between 2014–2015.	Maputo City residents, *n* = 656 In the latter survey	PVA OSP: cultivar NR	Frequency of consumption Annual meals with OSP Annual total urban consumption (tons)	[14]
Nigeria, rural	Building Nutritious Food Baskets (BNFB) project, a situation analysis in two phases: first, a desk review and content analysis; second, field visits and consultations with relevant stakeholders from 2015–2018	Farmers and consumers, *n* = 420 farmers and *n* = 735 consumers	PVA cassava: cultivar NR, released in 2011; PVA OSP: cultivar NR, released in 2012; PVA maize: cultivar NR, released in 2014	Current or ever consumption Frequency of consumption	[15]
Uganda, urban, per-urban	Consumer research, including study, design, testing, and implementation of behavioral interventions	Uganda: consumers, *n* = 122 interviews in 3 markets	Uganda: PVA OSP: cultivar NR	Ever consumption Frequency of purchase	[16]

Notes: NR, not reported; OSP, orange sweet potato; PVA, provitamin A.

**Table 4:** Characteristics of included studies for Impact Pathway 2, Indirect Consumption.

Country, Setting	Study Design	Population, *n*	Biofortified Crops Involved, Food Product or Processing	Outcomes Reported	Ref.
Mozambique, rural	Program evaluation in 2012, from 2006–2009 REU project	Women of reproductive age (*n* = 346) and children under 6 years of age (*n* = 178) in 36 villages	PVA OSP, cultivar: NR	Proportion households giving crop to neighbors by intervention group	[17]
Rwanda, rural	Impact assessment study, including a listing survey and a detailed household survey in 2015	Farmer households from the listing survey, *n* = 19,575 households in 120 randomly selected villages at the beginning of season B in 2015; plus 1397 bean-farming households	Iron bush beans released in 2010: RWR2445 RWR2154 MAC44 RWV1129 Year released: 2012 RWV3006 RWV3316 RWV2887 RWV3317 CAB2 MAC42	Current or ever consumption from gift or in-kind payment	[12]
Uganda, urban, per-urban	Consumer research, including study, design, testing, and implementation of behavioral interventions	Consumers, *n* = 122 interviews in 3 markets	PVA OSP: cultivar NR	Frequency of serving BF crops to children, spouses, other children, other adults	[16]

Notes: BF, biofortified; NR, not reported; OSP, orange sweet potato; PVA, provitamin A; REU, Reaching End Users.

**Table 5:** Characteristics of included studies for Impact Pathway 3, Formal Consumption.

Country, Setting	Study Design	Population, *n*	Biofortified Crops Involved, Food Product or Processing	Outcomes Reported	Ref.
Nigeria, rural	Intervention study in primary schools and follow-up surveys in 2016	Schoolchildren (7–12 y), *n* = 556 across 12 primary schools	PVA OSP, cultivar: NR	Average proportion of OSP in school meals	[18]
Brazil	Cross-sectional study in 3 rural public schools	Schoolchildren (5–12 y), *n* = 327	PVA OSP, cultivar: NR PVA cassava, cultivar: NR FeZn beans, cultivar: NR PVA maize, cultivar: NR	Amount (g) of each biofortified crop portion served to students as part of the school meal	[19]
6 regions including 85 countries, rural	Global Survey of School Meal Programs Report in 2019 *	Children (all ages), in 85 countries, *n* = 297.3 million receiving food through school meal programs	Any biofortified crops	Proportion of school lunch programs serving BF crops	[20]

Notes: BF, biofortified; NR, not reported; OSP, orange sweet potato; PVA, provitamin A. * A second survey round was repeated in 2021; results were not yet published at the time of writing this review.

**Table 6:** Characteristics of included studies for Impact Pathway 4, Farmer Household Consumption.

Country, Setting	Study Design	Population, *n*	Biofortified Crops Involved, Food Product or Processing	Outcomes Reported	Ref.
Mozambique, rural	Quasi-experimental study comparing 2-year intervention integrating agriculture and nutrition vs. control from 2003–2005 (informed the REU project, below)	Households, *n* = 741 from 3 districts	PVA OSP: Kandee, Japan, Lo, Taimung 64, Jonathan, CN, Resisto, Caromex, Cordner	Daily OSP consumption Prevalence of vitamin A deficiency (serum retinol < 0.7 µmol/L) Mean serum retinol Change in serum retinol	[21]
Reaching End Users (REU) project					
Mozambique, Uganda; rural	Technical report of the REU project, a clustered randomized trial using 2 dissemination strategies to increase OSP use, from 2006–2009	Uganda: Farmer households, *n* = 10,000 Mozambique: Farmer households, *n* = 12,000	PVA OSP: SPK 004 (Kakamega), Ejumula (Ejumula), SPK 004/6 (VITA), SPK 004/6/6 (Kabode), Cordner, Gabagaba, Jonathan, 0 5023 419, LO 323, MGCL 01, Resisto	Mean vitamin A intake OSP consumption Vitamin A intake from OSP	[22,23]
Mozambique, rural	Impact evaluation of the REU project from 2007–2009	Children (0–5 y), *n* = 781		OSP consumption as predictor of diarrhea incidence and severity	[24]
Mozambique, rural	Program evaluation of 2006–2009 REU project, 3 years after endline	Women of reproductive age (*n* = 346) and children under 6 years of age (*n* = 178) in 36 villages		Long-term: Mean vitamin A intake OSP consumption Vitamin A intake from OSP	[17]
Mozambique, Uganda; rural	Program evaluation of 2006–2009 REU project	Mozambique: Families with resident children between the ages of 6 and 35 months at the start of the study, *n* = 379 Uganda: Families with resident children between the ages of 36 and 71 months at baseline, *n* = 446		Variables mediating impact on vitamin A intakes by country	[25]
Mozambique, rural	Cluster-randomized controlled effectiveness study comparing two large-scale >2-y intervention programs (intensive inputs vs. reduced inputs) from 2006–2009	Children (6 mo to 5.5 y), *n* = 441 in 36 clusters; women, *n* = 441 in 36 clusters		Mean vitamin A intake OSP consumption Vitamin A intake from OSP	[26]
Uganda, rural	Follow-up analysis of the impact of the REU project on serum retinol	Children (6–35 mo), *n* = 264; children (3–5 y), *n* = 544; women, *n* = 539		Mean vitamin A intake OSP consumption Vitamin A intake from OSP Prevalence of vitamin A deficiency (serum retinol < 0.7 µmol/L) Prevalence of vitamin A (serum retinol < 1.05 µmol/L)	[27]
Uganda, rural	Impact evaluation, including gender roles and intra-household bargaining, from REU project from 2007–2009	Households, *n* = 775		Women’s bargaining power as a predictor of child’s OSP intake	[28,29]
Rwanda high iron beans project					
Rwanda, rural	Impact assessment study, including a listing survey and a detailed household survey in 2015 to examine impact of releasing high iron beans in 2010	Farmer households from listing survey, *n* = 19,575 households in 120 randomly selected villages in beginning of season B in 2015; plus 1397 bean-farming households; Farmer households; *n* = 422	Iron bush beans released in 2010: RWR2445 RWR2154 MAC44 RWV1129 Year released: 2012 RWV3006 RWV3316 RWV2887 RWV3317 CAB2 MAC42	Proportion of households that retain beans for their own consumption Frequency of consumption Consumption per capita	[12,30]
	Survey data from the 2015 impact assessment survey to investigate adoption	Farmer households: non-adopters, *n* = 971 adopters, *n* = 219	Fe common bean: CAB2, RWV3316, RWV3317, RWV3006, RWV2887, MAC44, MAC42	Effect of adoption on the duration of consumption from own production Effect of adoption on duration of market purchases	[31]
	Impact assessment study, including a listing survey and a detailed household survey in 2015 to examine the impact of releasing high-iron beans in 2010.	Farmer households from listing survey, *n* = 19,575 households in 120 randomly selected villages in beginning of season B in 2015; plus 1397 bean-farming households; Farmer households; *n* = 422	Bush beans: RWR2245	Average amount of beans consumed from own production per adult male equivalent Effect of growing RWR2245 on the length of time beans were consumed from own production	[32,33]
Reaching Agents of Change (RAC) project					
Tanzania, Mozambique, Nigeria, Ghana, Burkina Faso	RAC project, an ex-post survey from 2011–2015	Households, *n* = 309,974 (on track to benefit ≥600,000)	PVA OSP, cultivar: NR	Qualitative, frequency of OSP consumption, and amount of land devoted OSP cultivation	[34]
Building Nutritious Food Baskets (BNFB) project					
Nigeria, rural	BNFB project, a situation analysis in two phases: first, a desk review and content analysis; second, field visits and consultations with relevant stakeholders from 2015–2018	Farmers and consumers, *n* = 420 farmers and *n* = 735 consumers	PVA cassava: cultivar NR, released in 2011; PVA OSP: cultivar NR, released in 2012; PVA maize: cultivar NR, released in 2014	Reasons for cultivating BF crops Current or ever consumption	[15]
Sweetpotato Action for Security and Health in Africa project (SASHA); Marando Bora project					
Kenya, rural	Mama SASHA project, a cluster-randomized proof-of-concept from 2013–2018	Pregnant and lactating women followed through 9 months postpartum, *n* = 206	PVA OSP: Kabode, Vita	Frequency of consumption Contribution of OSP to vitamin A and beta-carotene intakes Odds of vitamin A deficiency (RBP < 1.17 µmol/L)	[35]
Tanzania, rural	Quasi-experimental field experiment, Marando Bora project, and survey data from 2010 and 2013	Farmer households, *n* = 434	PVA OSP: Kabode, Ejumula, Jewel	Proportion of OSP out of total sweet potato production	[36]
	Quasi-experimental field experiment, Marando Bora project, and survey data from 2010 and 2013	Farmer households, *n* = 919	PVA OSP: Kabode, Ejumula, Jewel	Qualitative, primary reason for growing OSP	[37]
Other projects					
Mozambique, urban	Analysis using several data sources including government statistical data, primary data on prices of sweet potato roots, semi-structured interviews, and a survey among Maputo City residents regarding production and consumption between 2014–2015	Maputo City residents, *n* = 656 In the latter survey	PVA OSP: cultivar NR	Frequency of consumption Annual meals with OSP Annual total urban consumption (tons)	[14]
Kenya, rural	Randomized controlled trial comparing agricultural training alone or in combination with nutrition and marketing training from 2015–2016	48 farmer groups, with 20–50 active members each	Fe/Zn Black beans: KK15	Qualitative, primary reason for growing BF beans	[38,39]
India, rural	Survey to investigate the value chain in 2016	4 villages, *n* = 310 farmers and consumers	PVA OSP, cultivar: NR	Proportion of farmers growing OSP Reasons when/why OSP is usually eaten	[40]
Guatemala, rural	Cluster-randomized trial comparing the distribution of BF or control bean seed, agronomic training, and nutrition information vs. control between 2015 and 2019.	Households (specifically adolescent girls) with bean production and high bean consumption, high prevalence of malnutrition and anemia, low presence of food aid programs, *n* = 1764 in 120 communities in 7 municipalities of 3 departments of eastern Guatemala	Fe/Zn black beans, cultivar: ICTA Chorti	Quantity of beans saved for consumption Quantity of beans consumed by girls in the last 24 h Amount of iron consumed per day from beans	[41] *

Notes: BF, biofortified; BNFB, Building Nutritious Food Baskets; NR, not reported; OSP, orange sweet potato; PVA, provitamin A; RAC, Reaching Agents of Change; RBP, retinol binding protein; REU, Reaching End Users; SASHA, Sweetpotato Action for Security and Health in Africa. 1Collected by the International Rice Research Institute in collaboration with the Department of Agricultural Extension (DAE) in Bangladesh. * Additional data via personal communication from Dr. Erick Boy.

## References

[1] Bailey R.L., West K.P., Black R.E. (2015). The epidemiology of global micronutrient deficiencies. Ann. Nutr. Metab..

[2] Beach R.H., Sulser T.B., Crimmins A., Cenacchi N., Cole J., Fukagawa N.K., Mason-D’Croz D., Myers S., Sarofim M.C., Smith M. (2019). Combining the effects of increased atmospheric carbon dioxide on protein, iron, and zinc availability and projected climate change on global diets: A modelling study. Lancet Planet Health.

[3] Welch R.M. (2005). Biotechnology, biofortification, and global health. Food Nutr. Bull..

[4] Council for Agricultural Science and Technology (CAST) (2020). Food Biofortification—Reaping the Benefits of Science to Overcome Hidden Hunger—A paper in the series on The Need for Agricultural Innovation to Sustainably Feed the World by 2050. Issue Paper 69.

[5] Strobbe S., Van Der Straeten D. (2018). Toward Eradication of B-Vitamin Deficiencies: Considerations for Crop Biofortification. Front. Plant. Sci..

[6] HarvestPlus (2018). Climate Change and Biofortification.

[7] HarvestPlus HarvestPlus Biofortified Crops Map and Table Updated with 2020 Data. https://www.harvestplus.org/knowledge-market/in-the-news/harvestplus-biofortified-crops-map-and-table-updated-2020-data.

[8] Blancquaert D., De Steur H., Gellynck X., Van Der Straeten D. (2014). Present and future of folate biofortification of crop plants. J. Exp. Bot..

[9] Global Alliance for Improved Nutrition (GAIN) and HarvestPlus (2020). The Commercialisation of Biofortified Crops Programme: Monitoring Reference Manual.

[10] Huey S.L., Krisher J.T., Friesen V., Mbuya M., Monterrosa E., Mehta S. (2021). Review of efficacy, effectiveness, and impact of biofortified foods and food products. Prospero.

[11] Page M.J., McKenzie J.E., Bossuyt P.M., Boutron I., Hoffmann T.C., Mulrow C.D., Shamseer L., Tetzlaff J.M., Akl E.A., Brennan S.E. (2021). The PRISMA 2020 statement: An updated guideline for reporting systematic reviews. BMJ.

[12] Asare-Marfo D.H., Herrington C., Birachi E., Birol E., Cook K., Diressie M.T., Dusenge L., Funes J., Katsvairo L., Katungi E. (2016). Assessing the Adoption of High-Iron Bean Varieties and Their Impact on Iron Intakes and Other Livelihood Outcomes in Rwanda: Main Survey Report.

[13] Petry N., Wirth J.P., Friesen V.M., Rohner F., Nkundineza A., Chanzu E., Tadesse K.G., Gahutu J.B., Neufeld L.M., Birol E. (2020). Assessing the Coverage of Biofortified Foods: Development and Testing of Methods and Indicators in Musanze, Rwanda. Curr. Dev. Nutr..

[14] Brouwer R., Tedesco I. (2019). Shackled Orange: Biofortified Varieties in the Sweetpotato Commodity Chain in Mozambique. Sustain. Agric. Res..

[15] Phorbee O., Mulongo G., Njoku N., Maru J., Munyua H. (2017). Baseline Report on Biofortification and Thematic Areas of Building Nutritious Food Baskets Project in Nigeria. (Nairobi, Kenya).

[16] Uchitelle-Pierce B., Ubomba-Jaswa P.A. (2017). Marketing biofortified crops: Insights from consumer research. Afr. J. Food Agric. Nutr. Dev..

[17] de Brauw A., Moursi M., Munhaua A.B. (2019). Vitamin A intakes remain higher among intervention participants 3 years after a biofortification intervention in Mozambique. Br. J. Nutr..

[18] Lagerkvist C.J., Carey E.E., Okello J.J., Kwikiriza N., Abidin P.E., Adekambi S. (2018). Goal-setting and volitional behavioural change: Results from a school meals intervention with vitamin-A biofortified sweetpotato in Nigeria. Appetite.

[19] Silva C.C.d.O., Deliza R., Nutti M.R., de Carvahlo J.L.V. (2015). Biofortificação de alimentos no município de Itaguaí: Melhorando a qualidade nutricional da merenda escolar (Biofortification in Itaguaí municipality: Improving nutritional quality of school meal). Reun. Biofortificação No Bras..

[20] The Global Child Nutrition Foundation (2019). Chapter 6: Health and Nutrition. The Global Survey of School Meal Programs.

[21] Low J.W., Arimond M., Osman N., Cunguara B., Zano F., Tschirley D. (2007). A food-based approach introducing orange-fleshed sweet potatoes increased vitamin A intake and serum retinol concentrations in young children in rural Mozambique. J. Nutr..

[22] Arimond M., Ball A.-M., Bechoff A., Bosch D., Bouis H., Brauw A., Coote C., Dove R., Eozenou P., Gilligan D. (2010). Reaching and Engaging End Users (REU) Orange Fleshed Sweet Potato (OFSP) in East and Southern Africa.

[23] HarvestPlus (2010). Disseminating Orange-Fleshed Sweet Potato. Findings from a HarvestPlus Project in Mozambique and Uganda.

[24] Jones K.M., de Brauw A. (2015). Using Agriculture to Improve Child Health: Promoting Orange Sweet Potatoes Reduces Diarrhea. World Dev..

[25] de Brauw A., Eozenou P., Gilligan D.O., Hotz C., Kumar N., Meenakshi J.V. (2018). Biofortification, crop adoption and health information: Impact pathways in Mozambique and Uganda. Am. J. Agric. Econ..

[26] Hotz C., Loechl C., de Brauw A., Eozenou P., Gilligan D., Moursi M., Munhaua B., van Jaarsveld P., Carriquiry A., Meenakshi J.V. (2012). A large-scale intervention to introduce orange sweet potato in rural Mozambique increases vitamin A intakes among children and women. Br. J. Nutr..

[27] Hotz C., Loechl C., Lubowa A., Tumwine J.K., Ndeezi G., Nandutu Masawi A., Baingana R., Carriquiry A., de Brauw A., Meenakshi J.V. (2012). Introduction of β-carotene-rich orange sweet potato in rural Uganda resulted in increased vitamin A intakes among children and women and improved vitamin A status among children. J. Nutr..

[28] Gilligan D.O., Kumar N., McNiven S., Meenakshi J.V., Quisumbing A. (2020). Bargaining power, decision making, and biofortification: The role of gender in adoption of orange sweet potato in Uganda. Food Policy.

[29] Gilligan D.O., Kumar N., McNiven S., Meenakshi J.V., Quisumbing A. (2014). Bargaining Power and Biofortification: The Role of Gender in Adoption of Orange Sweet Potato in Uganda. International Food Policy Research Institute (IFPRI)—Discussion Paper 01353.

[30] Asare-Marfo D., Herrington C., Alwang J., Birachi E., Birol E., Diressie M.T., Dusenge L., Funes J.E., Katungi E., Labarta R. (2016). Listing Exercise Report.

[31] Sellitti S., Vaiknoras K., Smale M., Jamora N., Andrade R., Wenzl P., Labarta R. (2020). The contribution of the CIAT genebank to the development of iron-biofortified bean varieties and well-being of farm households in Rwanda. Food Secur..

[32] Vaiknoras K., Larochelle C. (2021). The impact of iron-biofortified bean adoption on bean productivity, consumption, purchases and sales. World Dev..

[33] Vaiknoras K., Larochelle C. The Impact of Biofortified Iron Bean Adoption on Productivity, and Bean Consumption, Purchases and Sales. Proceedings of the 2018 Agricultural & Applied Economics Association Annual Meeting.

[34] Mulongo G., Munyua H., Mbabu A., Maru J. (2021). What is required to scale-up and sustain biofortification? Achievements, challenges and lessons from scaling-up Orange-Fleshed Sweetpotato in Sub-Sahara Africa. J. Agric. Food Res..

[35] Girard A.W., Grant F., Watkinson M., Okuku H.S., Wanjala R., Cole D., Levin C., Low J. (2017). Promotion of Orange-Fleshed Sweet Potato Increased Vitamin A Intakes and Reduced the Odds of Low Retinol-Binding Protein among Postpartum Kenyan Women. J. Nutr..

[36] Shikuku K.M., Okello J.J., Wambugu S., Sindi K., Low J.W., McEwan M. (2019). Nutrition and food security impacts of quality seeds of biofortified orange-fleshed sweetpotato: Quasi-experimental evidence from Tanzania. World Dev..

[37] Shikuku K.M., Okello J.J., Sindi K., Low J.W., McEwan M. (2019). Effect of farmers’ multidimensional beliefs on adoption of biofortified crops: Evidence from sweetpotato farmers in Tanzania. J. Dev. Stud..

[38] Ogutu S.O., Fongar A., Godecke T., Jackering L., Mwololo H., Njuguna M., Wollni M., Qaim M. How to Make Farming and Agricultural Extension More Nutrition-Sensitive: Evidence from a Randomized Controlled Trial in Kenya. Proceedings of the 30th International Conference of Agricultural Economists.

[39] Ogutu S.O., Fongar A., Godecke T., Jackering L., Mwololo H., Njuguna M., Wollni M., Qaim M. (2020). How to make farming and agricultural extension more nutrition-sensitive: Evidence from a randomised controlled trial in Kenya. Eur. Rev. Agric. Econ..

[40] Prakash P., Avinash K., Devesh R., Debdutt B., Sheela I. (2017). Biofortification for reducing hidden hunger: A value chain analysis of sweet potato in Odisha, India. Agric. Econ. Res. Rev..

[41] Reyes B., Mazariegos M., Maruyama E., Birol E., Boy E., Scollard P., Khor L.Y., Pérez S., Zeller M., González C. (2020). Socieconomic and Nutrition Impact of a Biofortified Bean Intervention in Adolescent Girls in Eastern Guatemala. PowerPoint Presentation.

[42] Sweetpotato Knowledge Portal HarvestPlus Reaching End Users Orange Fleshed Sweetpotato Project. https://www.sweetpotatoknowledge.org/project/harvestplus-reaching-end-users-orange-fleshed-sweetpotato-project/.

[43] Institute of Medicine (2006). Dietary Reference Intakes.

[44] Vaiknoras K., Larochelle C., Birol E., Asare-Marfo D., Herrington C. (2019). Promoting rapid and sustained adoption of biofortified crops: What we learned from iron-biofortified bean delivery approaches in Rwanda. Food Policy.

[45] Maziya-Dixon B.B., Akinyele I.O., Sanusi R.A., Oguntona T.E., Nokoe S.K., Harris E.W. (2006). Vitamin A deficiency is prevalent in children less than 5 y of age in Nigeria. J. Nutr..

[46] Cole D.C., Levin C., Loechl C., Thiele G., Grant F., Girard A.W., Sindi K., Low J. (2016). Planning an integrated agriculture and health program and designing its evaluation: Experience from Western Kenya. Eval. Program. Plann..

[47] McEwan M.A., Lusheshanija D., Shikuku K.M., Sindi K. (2017). Specialised Sweetpotato Vine Multiplication in Lake Zone, Tanzania: What “Sticks” and What Changes?. Open Agric..

[48] Walton J. (2019). Improving nutrition through biofortification: From strategy to implementation. Cereal Foods World.

[49] Walton J. Paving the Way to Commercialization: The Role of Standards for Biofortified Products. https://a4nh.cgiar.org/2021/12/22/paving-the-way-to-commercialization-the-role-of-standards-for-biofortified-products/.

